# Ultrasound Imaging of Cystic Nephroma

**DOI:** 10.15586/jkcvhl.2017.79

**Published:** 2017-07-20

**Authors:** Federico Greco, Eliodoro Faiella, Domiziana Santucci, Delia De Lisi, Gianguido Lo Vullo, Bruno Beomonte Zobel, Rosario Francesco Grasso

**Affiliations:** 1 Unit of Diagnostic Imaging, Università Campus Bio-Medico di Roma, Rome, Italy; 2 Medical Oncology Department, Università Campus Bio-Medico di Roma, Rome, Italy

**Keywords:** cystic nephroma, cystic renal cell carcinoma, DICER1, mixed epithelial stromal tumor, renal cystic lesions

## Abstract

Cystic nephroma is a rare, benign multicystic lesion of the kidney. This tumor occurs both in children and in adults. In children, it is highly prevalent in males; in adults, it is more frequent in women. The term “cystic nephroma” represents two apparently different entities: pediatric cystic nephroma, a benign form thought to originate from metanephric tissue, and adult cystic nephroma, considered as a lesion of mixed epithelial stromal tumor. The clinical presentation may be a palpable mass or nonspecific symptoms such as abdominal pain, hematuria, and urinary tract infections. In this review, we summarize the ultrasound imaging features of cystic nephroma and describe the characteristics of the most common renal cystic lesions and the differential diagnosis of cystic nephroma with other renal cystic lesions.

## Introduction

Cystic nephroma (CN) is a rare, benign multicystic lesion of the kidney (1). This tumor has two peaks of distribution: two-thirds occur in children between 3 months and 2 years, with a higher prevalence in males; and one-third occurs in adults over the age 30, with a female predominance. About 5% of this rare lesion appears in patients between 5 and 30 years of age (2). The term CN has been used to refer two apparently different lesions (3). The first type, pediatric CN, originates from the metanephric tissue and presents benign characteristics (4, 5). The second type, adult CN, occurring mainly in women, is considered as cystic lesion of mixed epithelial stromal tumor (MEST) (6–9). Germline mutations of DICER1 cause DICER1 syndrome. This syndrome causes different types of tumors, including CN, in children and young adults. Other tumors of DICER1 syndrome are Sertoli–Leydig cell tumors, renal sarcoma, rhabdomyosarcoma, pineoblastoma, pituitary blastoma, and medulloepithelioma (10–14).

**Figure 1:**
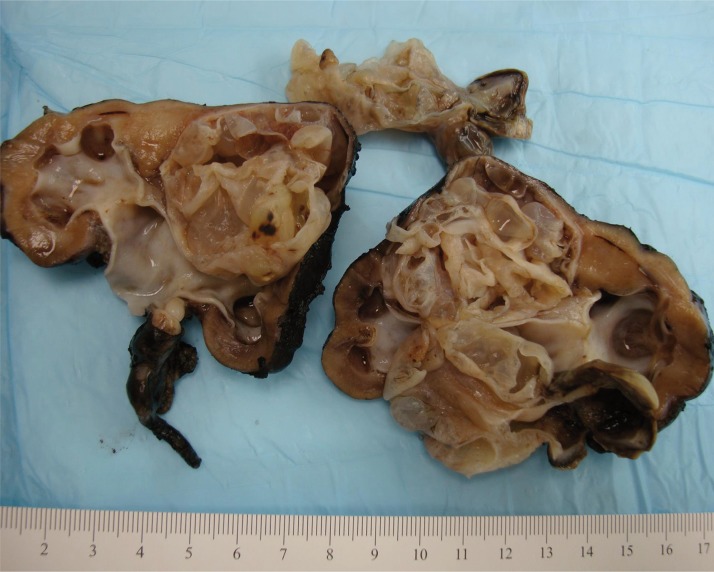
Macroscopic examination of bilateral CN of a 5-month-old male. Image provided by Professor Ellen M. Chung and Director Donald E. Hatley Jr.

At macroscopic examination, CN and cystic partially differentiated nephroblastoma (CPDN) are indistinguishable. They are typically unifocal and rarely multifocal or bilateral. Lesion dimensions range between 5 and 10 cm and are surrounded by a thick fibrous capsule and compressed parenchyma. The loculi are between a few millimeters and 4 cm in diameter, filled with colorless fluid or thick myxoid material and separated by thin fibrous septa (Figure 1). The tumor may herniate inside the collecting system. On microscopic examination, the septa are formed by aligned hobnail, cuboid, or flat cells. CN and CPDN can be distinguished microscopically because CN has mature tubules between the septa, while the CPDN presents blastemal cells and/or other embryonal elements. Nodules of blastemal cells would lead to a diagnosis of cystic nephroblastoma (5, 15–17).

A recent study showed genetic, immunohistochemical, and morphological differences between pediatric CN and adult CN/MEST (18). Pediatirc CN has a higher prevalence of DICER1 mutations. Wavy and ropy collagen, which is typical of adult CN/MEST, is not found in pediatric CN. Immunostaining for inhibin is absent in pediatric CN, while it is present in adult CN/MEST. Finally, estrogen receptor immunoreactivity and cellular stroma are present in both pediatric CN and adult CN/MEST. In summary, the stroma of adult CN/MEST, unlike pediatric CN, has the phenotype of ovarian stroma (18). The detected differences justify the distinction of pediatric CN and adult CN/MEST as two independent entities.

The presentation of the lesion may be a palpable mass without any symptoms or with nonspecific symptoms such as abdominal pain, hematuria, and urinary tract infection (19). Ultrasound (US) is the first choice for the evaluation of kidney lesions. Cystic renal lesions, both benign and malignant, display similar characteristics on US, and hence, differential diagnosis is often difficult. In this review, we describe US imaging features of CN based on recent studies. Knowledge of the CN characteristics is important for differential diagnosis with other renal cystic lesions.

A PubMed search was performed for the term “cystic nephroma.” Articles in English language, published in the last 5 years, describing US imaging of CN were selected. Articles with coarse description of CN imaging were not selected.

## Ultrasound of cystic nephroma

Ultrasound (US) examination of the kidney is performed using a curvilinear transducer with frequencies ranging from 2.5 to 5.0 MHz. The echogenicity of the normal renal cortex should be medium-level gray, slightly lower compared to echogenicity of the liver. The renal pyramids appear as hypoechoic, while the central renal sinus is echogenic. The renal artery and vein are identified at the level of the renal hilum and their visualization improves with color Doppler or power Doppler. Typically, normal ureters are not visible during US (20).

There have been several cases of CN investigated by US. Wani et al. reported a case of an 11-month-old male infant with asymptomatic right abdominal mass. US examination identified a retroperitoneal multilocular, avascular, rounded cystic mass in the kidney area. The right kidney was not visible (21). Bagley et al. (20) reported a case of a 66-year-old man with symptoms suggestive of kidney stones. The patient underwent computed tomography (CT) and magnetic resonance imaging (MRI) examinations. Subsequently, biopsy revealed the presence of CN. US examination showed the presence of multiple, small, simple cysts localized in the inferior pole of the right pelvic kidney (right pelvic kidney identified previously through CT examination). Left kidney was located superiorly in relation to the normal anatomical position and measured 11.8 cm in longitudinal diameter, 6.7 cm in transverse diameter, and 5.1 cm in anteroposterior diameter. CN was localized at the level of the lateral portion of the upper pole of the left kidney and was well defined with thick septa and posterior enhancement. The dimensions of the CN were 6 cm in longitudinal diameter, 5.7 cm in transverse diameter, and 4.6 cm in anteroposterior diameter. Color Doppler did not show flow within the mass (20). Wilkinson et al. presented six adult patients (five females and one male) with CN. All patients had loin pain, two patients had hematuria, two patients presented with recurrent urinary infections, and one patient reported significant loss of weight. US was performed in five patients, which showed the following findings: a complex cyst of 7 cm at the mid pole of the left kidney, a multiloculated cyst at the upper pole of the right kidney, a large cyst of 10 cm at the upper pole of the right kidney, a complex cyst of 7 cm at the upper pole of the right kidney, and a complex cyst of 5.8 cm in diameter at the left kidney (22).

Kousari et al. (23) reported a case of DICER1 syndrome in a 5-month-old full-term female infant. This infant presented with fever and tachypnea. She had a history of persistent tachypnea, grunting, irregular wheezing since birth, and episodic nonproductive cough. The result of a chest radiograph prompted to carry out an unenhanced chest CT. It showed a large cystic lesion with thin internal septa localized in the upper lobe of the left lung, which determined mass effect and contralateral mediastinal shift. Renal US showed a normal right kidney and an anechoic lesion with a diameter of 7 mm without internal vascularization at the lower pole of the left kidney. At 5-month follow-up, US showed an increase in size of the cystic lesion and the presence of a second complex cyst with internal septa on the same kidney. At the age of five, a left ovarian torsion, due to a poorly differentiated Sertoli cell tumor, was diagnosed (23). Aparanji et al. (24) reported a case of an 11-month-old infant boy with two bilateral abdominal masses, developed in a month, and abdominal distension. Laboratory studies detected a serum creatinine level of 0.52 mg/dL and a glomerular filtration rate of 60 mL/min/1.73 m^2^ calculated with the Schwartz formula. US of the kidney revealed the presence of bilateral, large, multiloculated, well-demarcated cystic renal masses (24). A further clinical case of a 48-year-old man was presented by Pastor Navarro et al. (25). This patient suffered from primary biliary cirrhosis and presented with hematuria, febrile syndrome, and recidivant lower respiratory tract disease. US of the kidney detected a multilobular lesion at the lower pole of the right kidney, with echogenic content of 3 × 3 cm (25).

Dong et al. (26) described a single case of a 30-year-old man hospitalized for a 2-year history of intermittent pain in the right flank and gross hematuria. Renal US identified a complex cystic mass at the upper pole of the right kidney, with maximum dimension of 6.5 cm in axial diameter (26). Ozturk and Karaaslan (27) reported a case of CN in a 59-year-old female who presented with left flank pain and abdominal pain. A mobile and dolorous mass was detected with palpation in the left lumbar region. US showed multilocular renal cysts with hyperechoic septa and semisolid aspect (size of 22 × 10.9 × 8.2 cm). MR exam showed multilocular cystic renal mass of the left kidney, hypointense on T1-weighted images and hyperintense on T2-weighted images (size of 24.5 × 11.9 × 9.8 cm), and a multicystic aspect in ureter projection, with the largest portion 1.7 cm in diameter. Imaging features led to the suspicion of multicystic renal cell carcinoma (RCC) or multilocular CN. After nephroureterectomy, macroscopic examination revealed the presence of multiple cysts tensely adherent to the internal wall of the ureter. Microscopic examination showed cuboidal and hobnail cells of the epithelial ovarian-type stroma and areas composed of occasional fusiform cells. This was the first case of multilocular CN with uretheral invagination described in the literature (27).

Karmazyn et al. (28) described the US images of a 7.2-year-old boy who presented with gross hematuria. The US showed a multilocular cystic mass with vascularity of cystic walls and septa. Histopathological diagnosis, after radical nephrectomy, revealed the presence of multiloculated CN (28). Kurian et al. (29) presented a case of a 4-month-old male child with a right flank mass, progressively increasing in size over 2 months. Examination showed a mobile, soft, cystic, and bimanually palpable mass, while the left flank was clinically normal. US and CT detected two well-demarcated multilocular cystic lesions, the largest localized at the level of the middle/lower pole of the right kidney, and another smaller lesion at the upper pole of the left kidney. Imaging features were suggestive of bilateral CN/bilateral CPDN. After bilateral partial nephrectomy and histological analysis, the diagnosis of bilateral CN was made. After 2 years, the patient developed a polypoid mass protruding from the external urethral meatus. Histological examination revealed the presence of embryonal rhabdomyosarcoma. After disease staging (stage 1), the patient was treated with complete excision of the penile urethra and postoperative chemotherapy with eight cycles of vincristine, actinomycin-D, and ifosfamide. The urethra was rebuilt with a dorsal island prepucial flap urethroplasty. The association of CN and rhabdomyosarcoma could be related to DICER1 mutation (in this case, the genetic analysis was not performed) (29).

Dell’Atti (30) showed a case of a 66-year-old man with dysuria, urinary frequency, and recent *Escherichia coli* urinary tract infection, despite 2 weeks of therapy with ciprofloxacin. The patient presented with a slight knocking pain in the left kidney zone at physical examination and microscopic hematuria at laboratory examination. A well-circumscribed cystic mass of the left kidney (maximum diameter of about 4 cm), with thickened walls and a hyperechoic aspect (large calcifications) at the middle third of the kidney, was revealed during renal US examination. Subsequently, CT examination revealed mass characteristics suggestive of malignancy and was categorized as Bosniak cyst type 3. After laparoscopic nephrectomy, macroscopic and microscopic histopathological examinations led to a diagnosis of CN. This is an unusual case because of the presence of a single cyst with calcified walls and small loculi divided by thin septa (30). Mandal et al. (31) retrospectively evaluated 15 cases of uncommon renal tumors in pediatric population. There were four cases of clear cell sarcoma, three cases of rhabdoid tumor, three cases of congenital mesoblastic nephroma, two cases of multilocular CN, two cases of renal teratoma, and one case of teratoid Wilms’ tumor. In the two cases of multilocular CN, a mass at the lumbar level and hematuria were discovered. In both patients, a cystic lesion with clear demarcation from the renal parenchyma was shown at renal US examination. Contrast-enhanced CT confirmed the presence of multilocular cystic lesions that appeared well circumscribed, with thickened walls, and without the evidence of calcification or contrast enhancement. After nephrectomy, histopathological examination confirmed the cyst as CN (31). Faure et al. (32) described two cases of pediatric patients with CN. The first case was a 4-year-old male patient with a multiloculated mass of the right kidney of 9 × 9 × 6.9 cm diameter at US and CT examinations, with septa inside, compatible with CN or CPDN. After right heminephrectomy, histopathological examination confirmed the diagnosis of CN. DICER1 testing of the father, proband, and sibling presented a heterozygous loss-of-function mutation. The second case was a 5-year-old girl with diagnosis of Sertoli–Leydig cell tumor with a cystic renal lesion localized in the right kidney with the size of 2.8 × 2.3 × 2 cm at US and CT examinations. After right heminephrectomy, histopathological examination demonstrated the presence of CN. A DICER1 mutation was detected (32). Chung et al. (33) in describing the radiological-pathologic correlations of renal tumors in childhood showed the US images of an 11-month-old girl. In this case, the lesion appeared as a complex mass with many internal cysts localized in the lower pole of the left kidney. This mass showed caliectasis and some flows at the level of the septa during the color Doppler study (33).

**Figure 2:**
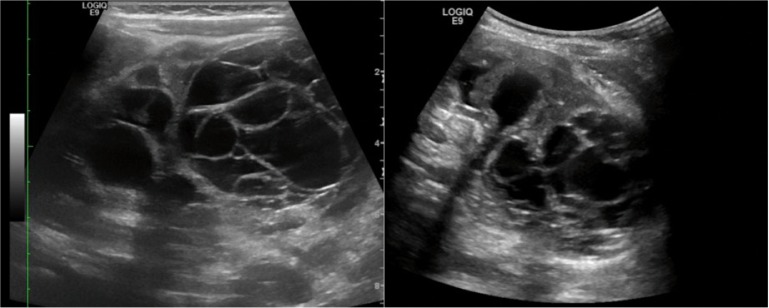
Renal US of a 5-month-old male (same patient of Figure 1). The images show the presence of bilateral CN that appears as multiloculated cystic lesions, with margins well circumscribed by the surrounding renal parenchyma and internal septa. Caliectasies are also present. Image provided by Professor Ellen M. Chung and Director Donald E. Hatley Jr.

We analyzed 20 cases of CN described in the recent literature (10 pediatric CN and 10 adult CN) (20–33). In the 10 pediatric cases, 13 lesions were evaluated. Among these lesions, five were located in the right kidney, five in the left kidney, and in three, localization of the lesions was not specified. Furthermore, 2 of these 10 patients had bilateral lesions, 1 showed vascularity of the cystic wall and septa at color Doppler. Another lesion caused caliectasis and showed some flow within the septa at color Doppler (21, 23, 24, 28, 29, 31–33) (Table 1). In the 10 adult CNs described, 5 were located in the right kidney and 5 in the left kidney (1 with uretheral invagination and 1 with lesion calcifications) (20, 22, 25–27, 30) (Table 2). On US imaging, they appeared as multiloculated cystic lesions, with edges well circumscribed by the surrounding renal parenchyma and septa inside (Figure 2). Vascularity, calcifications, and caliectasis were rare.

**Table 1:** US features of pediatric CN

References	Right kidney	Left kidney	Caliectasis	Calcifications	Vascularity
Wani et al. (21)	1	-	-	-	-
Kousari et al. (23)	-	2	-	-	-
Aparanji et al. (24)	1	1	-	-	-
Karmazyn et al. (28)	Not specified	Not specified	-	-	1
Kurian et al. (29)	1	1	-	-	-
Mandal et al. (31)	Not specified	Not specified	-	-	-
Faure et al. (32)	2	-	-	-	-
Chung et al. (33)	-	1	1	-	1

**Table 2:** US features of adult CN

References	Right kidney	Left kidney	Caliectasis	Calcifications	Vascularity
Bagley et al. (20)	-	1	-	-	-
Wilkinson et al. (22)	3	2	-	-	-
Pastor Navarro et al. (25)	1	-	-	-	-
Dong et al. (26)	1	-	-	-	-
Ozturk et al. (27)	-	1	-	-	-
Dell’Atti et al. (30)	-	1	-	1	-

## Diagnostic criteria

Powell et al. (34) have proposed the following criteria to diagnose CN:

- Multilocular lesion
- Unilateral lesion
- Noncommunication of the cyst with the renal pelvis
- Noncommunication of the cysts with each other
- Loculi aligned by epithelium
- Intralocular septa devoid of renal parenchyma
- If residual renal tissue is present, it should be normal

Subsequently, Boggs and Kimmelstiel (35) modified these criteria, to include the presence of immature renal tissue in the septa.

Finally, the diagnostic criteria were revised by Joshi and Beckwith (5) as follows:

- The entire lesion is composed of cysts of various sizes and by septa that separate the cysts.
- Cystic lesion is clearly distincted from the normal renal parenchyma.
- The only solid component of the cysts is the septa.
- Cysts are aligned by cuboidal or hobnail cell epithelium.
- The septa are composed of fibrous tissue and can include well-differentiated renal tubules (Table 3).

## Differential diagnosis

**Figure 3:**
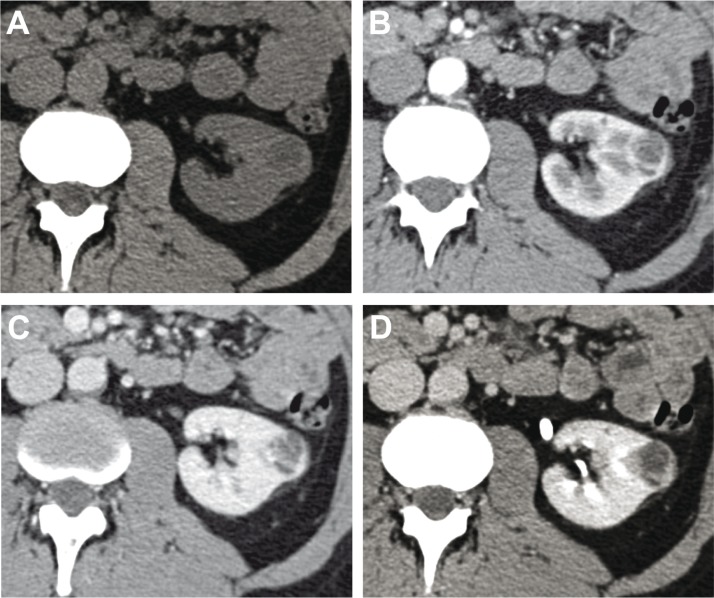
Unenhanced CT axial scan (a), CT axial scan during arterial (b), venous (c), and nephrographic (after 180 seconds from the contrast medium injection) (d) phases of a 44-year-old male, showing the presence of an oval, partially esophytic lesion with predominantly fluid-corpusculated content and internal septa. Cytological and histological examination have placed the diagnosis of clear cell renal cell carcinoma.

**Figure 4:**
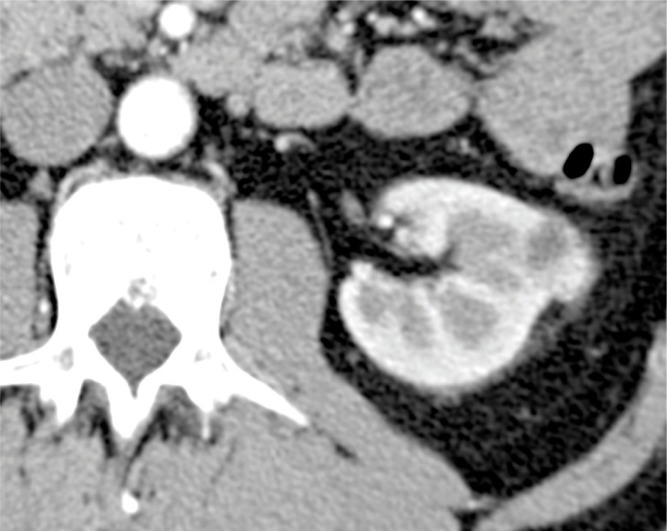
CT axial scan during arterial phase of a 44-year-old male (same patient of Figure 3) shows a minute hypervascular solid component located in the cranial portion of the clear cell renal cell carcinoma.

Differential diagnosis of CN or MEST with other renal cystic lesions, especially nephroblastoma and cystic renal cell carcinoma (CRCC) (19, 36), is challenging because of similar imaging features (37). Differential diagnosis is essential because prognosis of these lesions changes (20). CN and MEST have very similar diagnostic imaging features. However, unlike CN, in MEST, there is a greater likelihood of finding smaller cysts, thicker septa, solid components, and vascularization of septa (38). The classic CT characteristic of MEST is a well-circumscribed, multiseptated, delayed contrast-enhanced cystic solid mass. At MRI, the MEST cystic region appears hypointense on T1-weighted and hyperintense on T2-weighted sequences, while the central component appears as a nodular lesion hyperintense on T1-weighted and hypointense on T2-weighted sequences, and shows contrast enhancement (39). Nephroblastoma can be misinterpreted as CN because it may contain cystic components. These components, however, are more tightly associated with necrosis or hemorrhage. On US imaging, nephroblastoma has the same echogenicity of the liver and contains a greater solid component than CN (40), while CT reveals a heterogeneous, large, intrarenal often solid mass (33). It can also have a macrolobulated aspect, nodules satellites, and ellipsoidal or semilunar hyperdensity, representatives of compressed residual renal parenchyma (33, 41). Unlike CN, nephroblastoma could infiltrate the renal vein or inferior vena cava (42, 43). On CT examination, calcifications are seen in roughly 15% of nephroblastoma (44, 45). At MRI, nephroblastoma appears heterogeneous, lobulated, and hypointense on T1-weighted compared with healthy renal parenchyma and hyperintense or isointense on T2-weighted sequences. After injection of the contrast medium, nephroblastoma shows less contrast enhancement than the adjacent kidney (46). CRCC involves two forms of RCC: the cystic clear-cell carcinoma and a rare form of neoplasia, called multilocular CRCC (47–50). CRCC is very difficult to differentiate from CN using US, but the presence of intramural nodules and thick septa is suggestive of CRCC. At CT and MRI, CRCC appears as unilocular or multilocular lesion, with water-attenuating signal, irregular walls, and septa that show enhancement after contrast medium injection (50, 51). Other features that may be present are asymmetric enhancement of the walls and septa, and calcifications of the walls (52). Another CT sign of malignancy is an increase in density greater than 20 HU before contrast medium injection, and a further increase in density greater than 10 HU after contrast medium injection (53). CRCC rarely appears as a solid mass (50) (Figures 3 and 4).

**Table 3:** Diagnostic criteria of CN

DIAGNOSTIC CRITERIA
Powell et al. (34)
Multilocular lesion
Unilateral lesion
Non-communication of the cyst with the renal pelvis
Non-communication of the cysts with each other
Loculi aligned by epithelium
Intralocular septa devoid of renal parenchyma
Boggs and Kimmelstiel (35)
Multilocular lesion
The cyst must, for the most part, be lined by epithelium
Non-communication of the cyst with the renal pelvis
The residual renal tissue should be essentially normal, except for pressure atrophy
Presence of immature renal tissue in the septa
Joshi and Beckwith (5)
The entire lesion is composed of cysts of various sizes and by septa that separate the cysts
Cystic lesion is clearly distincted from the normal renal parenchyma.
The only solid component of the cysts are the septa
Cysts are aligned by cuboidal or hobnail cell epithelium.
The septa are composed of fibrous tissue and can include well-differentiated renal tubules

## Conclusion

CN is a rare, benign renal neoplasm, which can be distinguished into two forms: pediatric CN and adult CN/MEST. A summary of US imaging studies of CN, published in the last 5 years, shows that CN presents as multiloculated lesions, well circumscribed by the surrounding renal parenchyma with thin septa inside, and without evidence of vascularity or calcifications. Uncommon characteristics of pediatric CN were the presence of vascularity of the walls and septa at color Doppler, caliectasis, and some flow within the septa at color Doppler. Rare features of adult CN were uretheral invagination and calcifications of the wall. Differential diagnosis of other renal cystic lesions with CN or MEST by imaging could be challenging. This is an area that requires further research.

## Conflict of interest

The authors declare no potential conflicts of interest with respect to research, authorship, and/or publication of this article.
